# Supplementary figures and images for: Long non‑coding RNA L13Rik promotes high glucose-induced mesangial cell hypertrophy and matrix protein expression by regulating miR-2861/CDKN1B axis
Source: PeerJ. 2023 Oct 16;11:e16170. doi: 10.7717/peerj.16170 (PMC10586299; doi:10.7717/peerj.16170)

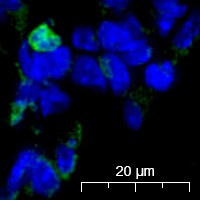

Supplement: Supplemental Information 1 [file peerj-11-16170-s001.zip › Figure 1 raw data/Figure 1F raw image/HG-20um.jpg]

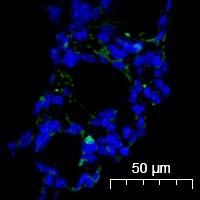

Supplement: Supplemental Information 1 [file peerj-11-16170-s001.zip › Figure 1 raw data/Figure 1F raw image/HG-50um.jpg]

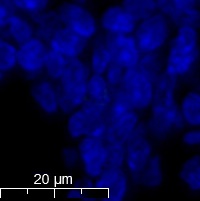

Supplement: Supplemental Information 1 [file peerj-11-16170-s001.zip › Figure 1 raw data/Figure 1F raw image/NG-20um.jpg]

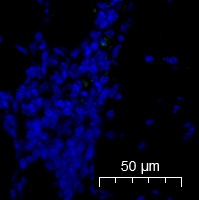

Supplement: Supplemental Information 1 [file peerj-11-16170-s001.zip › Figure 1 raw data/Figure 1F raw image/NG-50um.jpg]

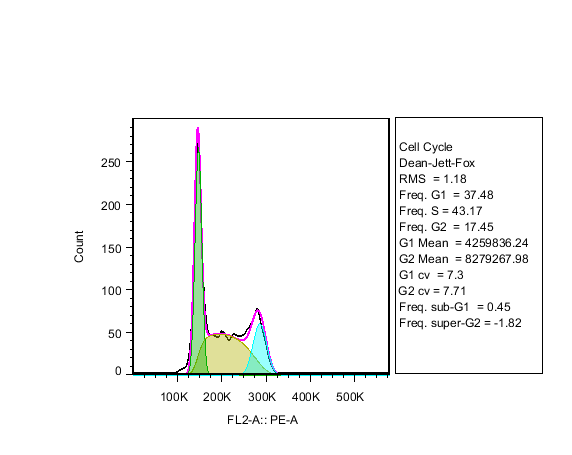

Supplement: Supplemental Information 1 [file peerj-11-16170-s001.zip › Figure 2 raw data/Figure 2-flow cytometry/A1.png]

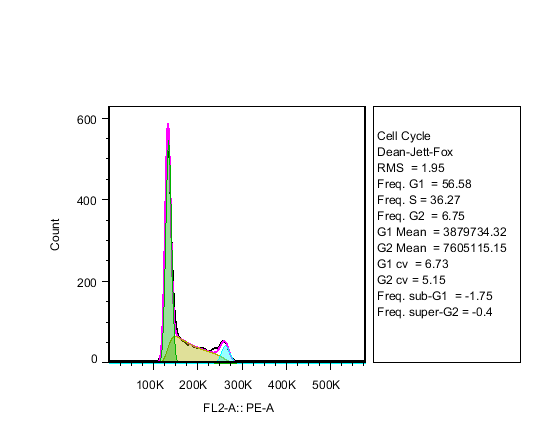

Supplement: Supplemental Information 1 [file peerj-11-16170-s001.zip › Figure 2 raw data/Figure 2-flow cytometry/B1.png]

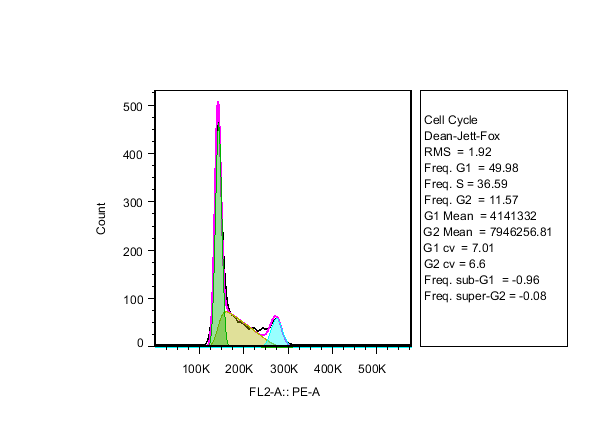

Supplement: Supplemental Information 1 [file peerj-11-16170-s001.zip › Figure 2 raw data/Figure 2-flow cytometry/C1.png]

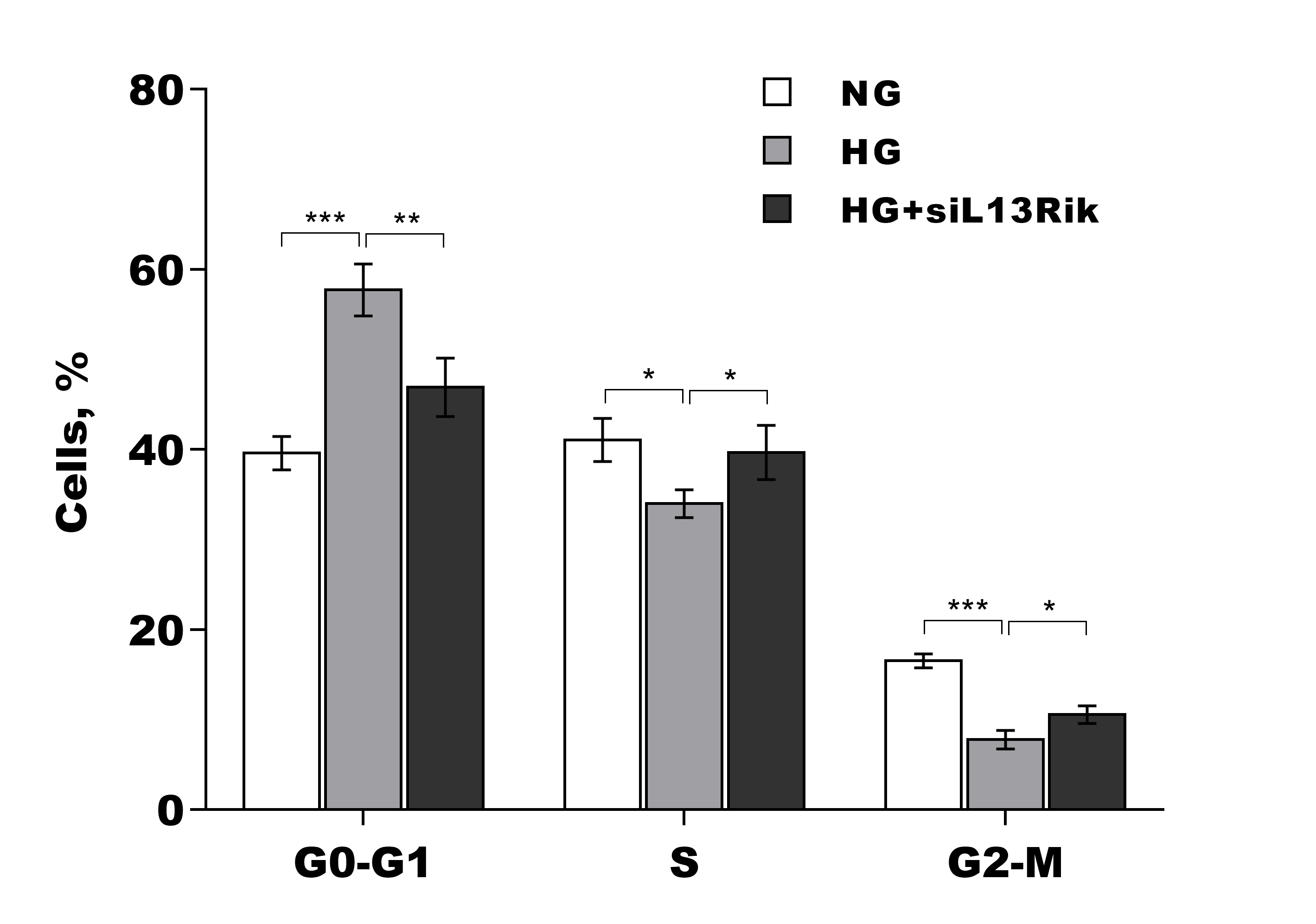

Supplement: Supplemental Information 1 [file peerj-11-16170-s001.zip › Figure 2 raw data/Figure 2-flow cytometry/fig 2.tif]

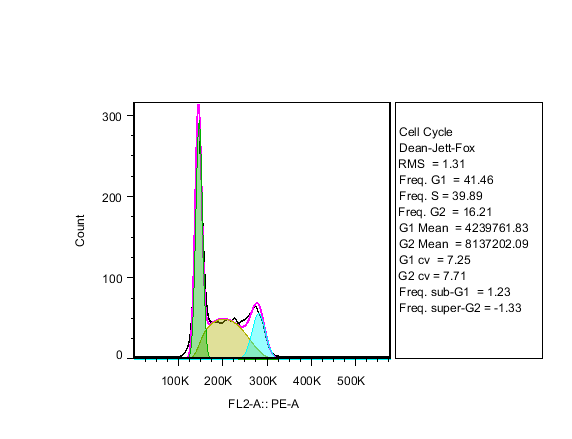

Supplement: Supplemental Information 1 [file peerj-11-16170-s001.zip › Figure 4 raw data/Figure 4-flow cytometry/A2.png]

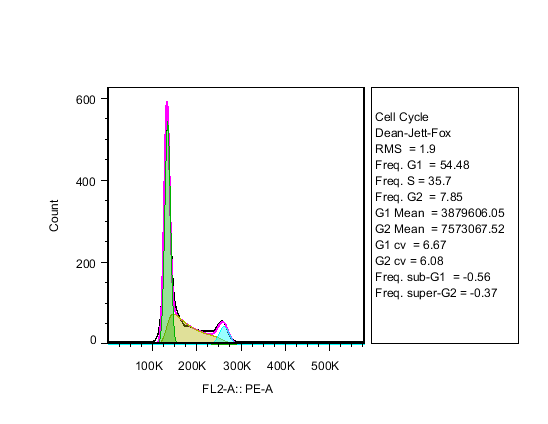

Supplement: Supplemental Information 1 [file peerj-11-16170-s001.zip › Figure 4 raw data/Figure 4-flow cytometry/B2.png]

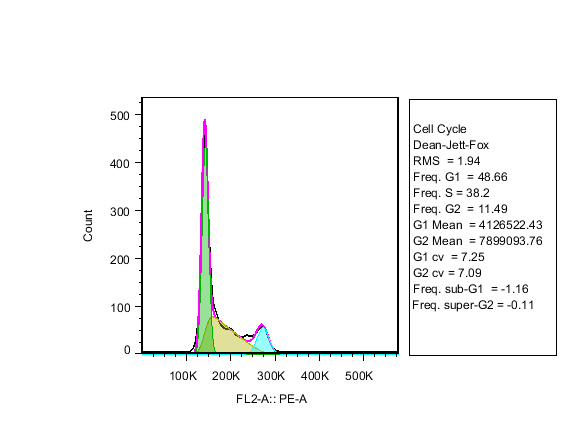

Supplement: Supplemental Information 1 [file peerj-11-16170-s001.zip › Figure 4 raw data/Figure 4-flow cytometry/C2.png]

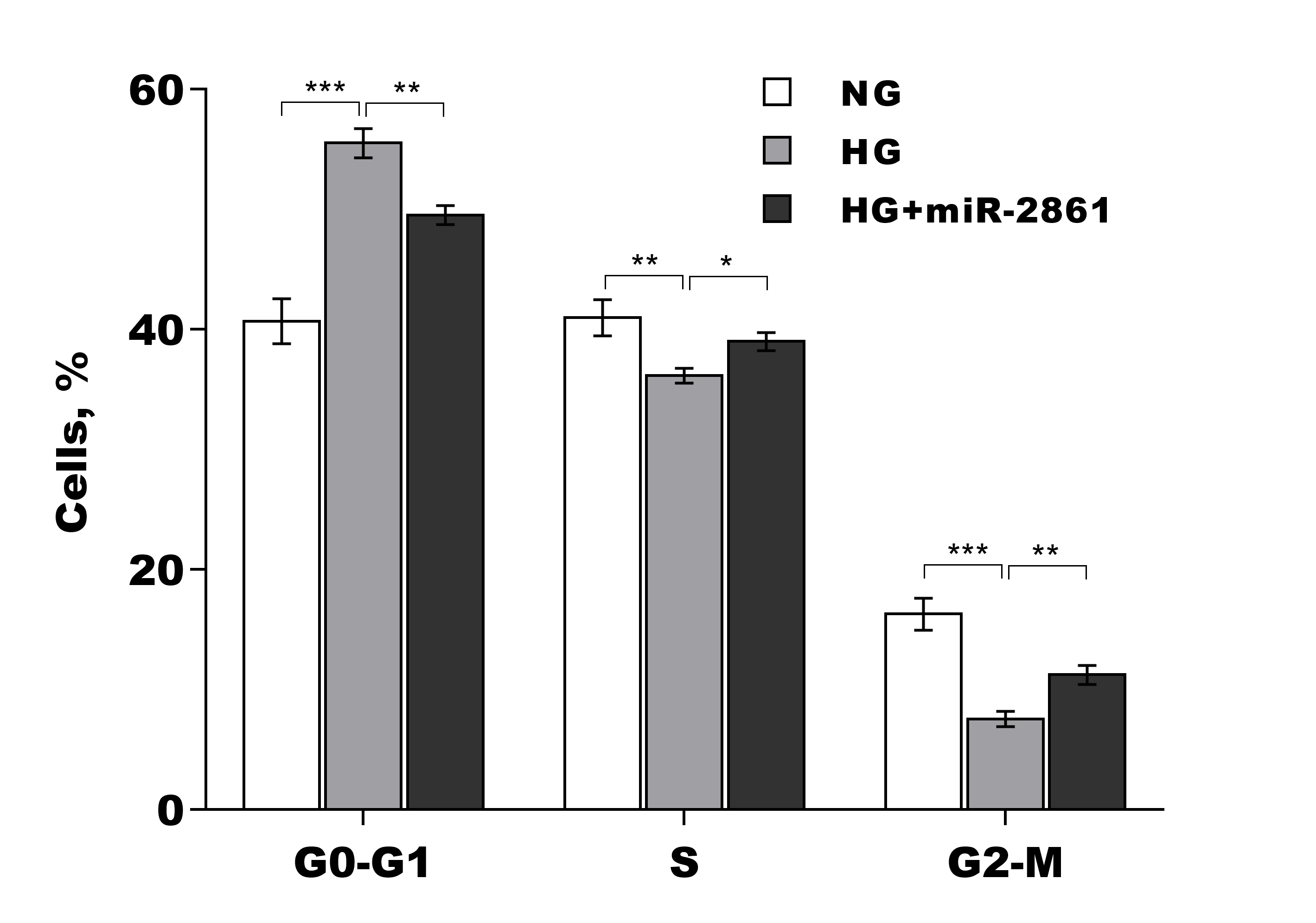

Supplement: Supplemental Information 1 [file peerj-11-16170-s001.zip › Figure 4 raw data/Figure 4-flow cytometry/fig 4.tif]

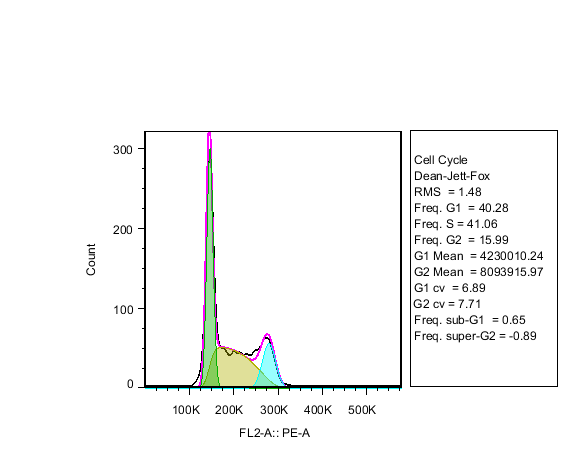

Supplement: Supplemental Information 1 [file peerj-11-16170-s001.zip › Figure 5 raw data/Figure 5-flow cytometry/3afe0d19670e0466531f406af8a896b.png]

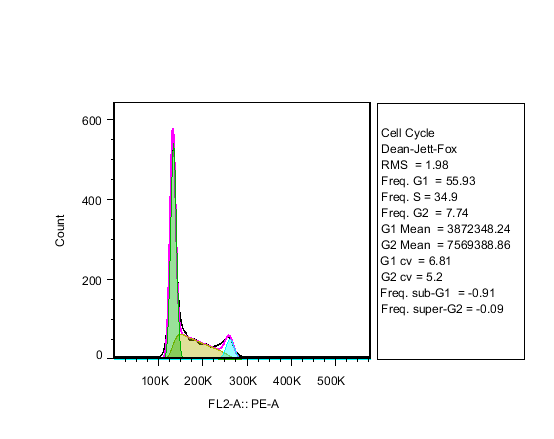

Supplement: Supplemental Information 1 [file peerj-11-16170-s001.zip › Figure 5 raw data/Figure 5-flow cytometry/78e6ac02dba479c255f9cc4efd90155.png]

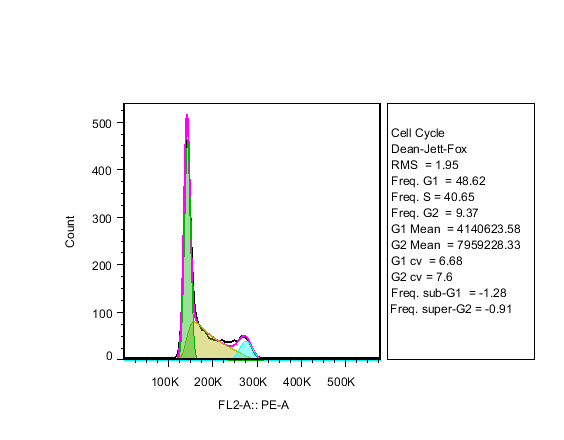

Supplement: Supplemental Information 1 [file peerj-11-16170-s001.zip › Figure 5 raw data/Figure 5-flow cytometry/8bcc86453add4fab407e924cace732d.png]

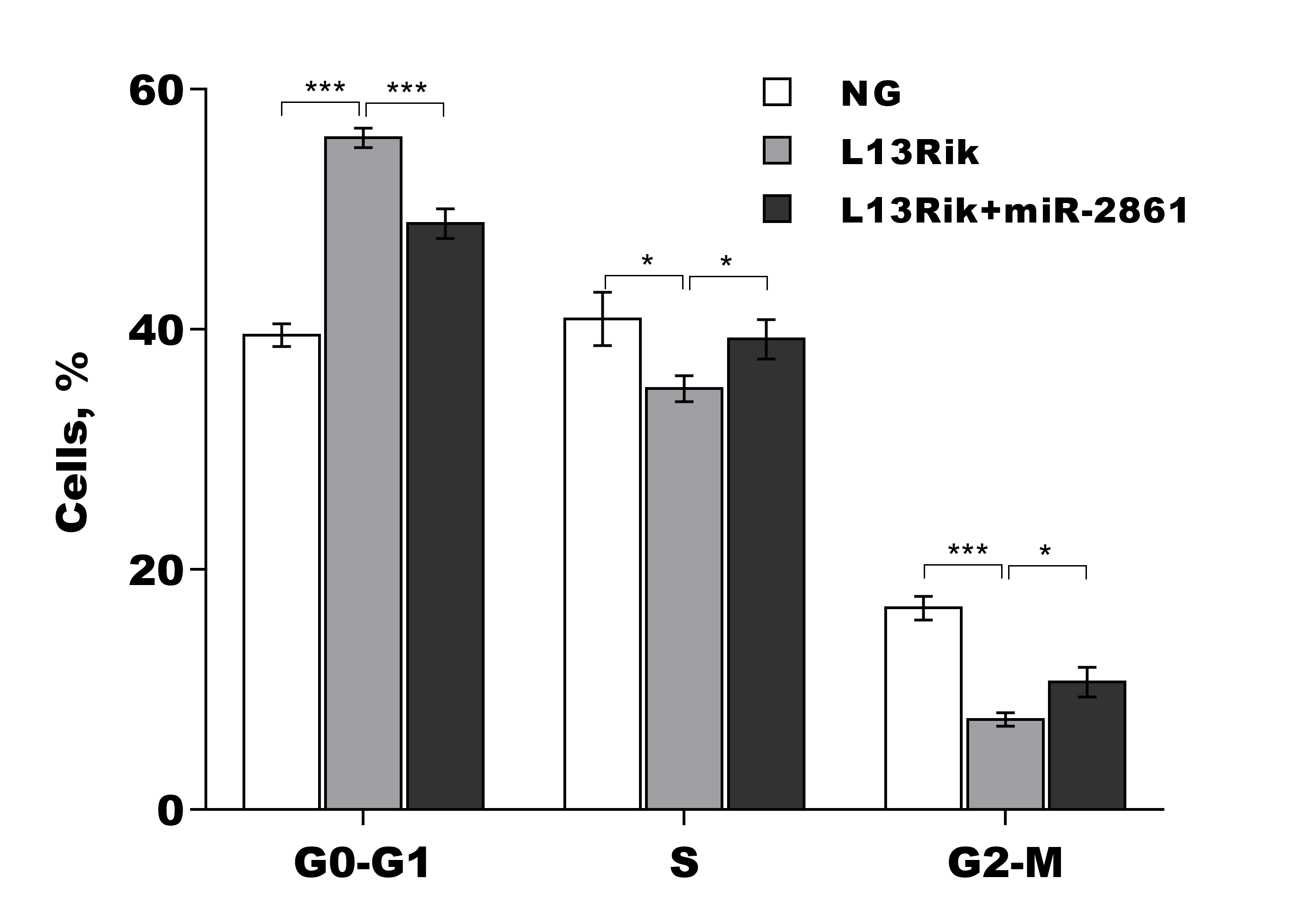

Supplement: Supplemental Information 1 [file peerj-11-16170-s001.zip › Figure 5 raw data/Figure 5-flow cytometry/fig 5.tif]

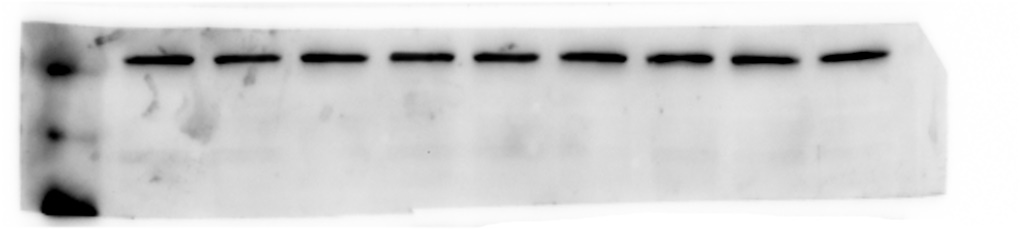

Supplement: Supplemental Information 1 [file peerj-11-16170-s001.zip › WB picture/Figure 2-4-5/Fig 2,4,5-Actin.jpg]

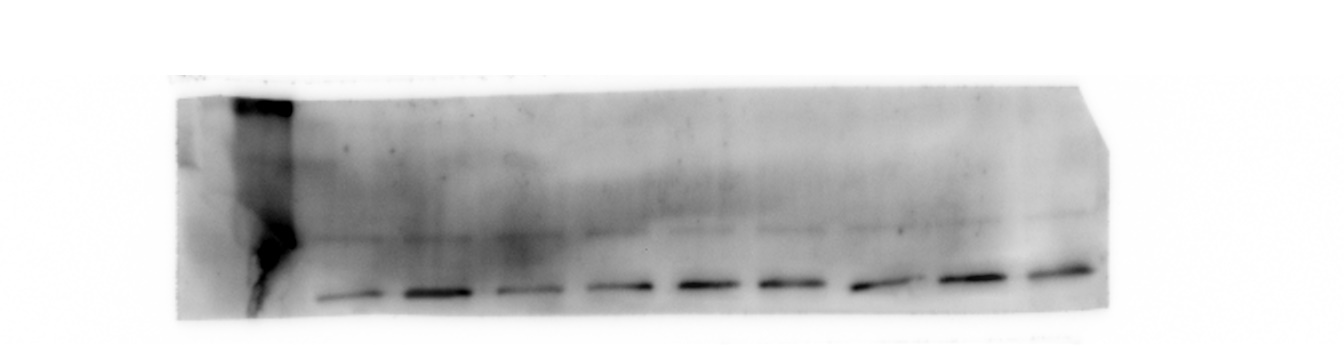

Supplement: Supplemental Information 1 [file peerj-11-16170-s001.zip › WB picture/Figure 2-4-5/Fig 2,4,5-N-cad.jpg]

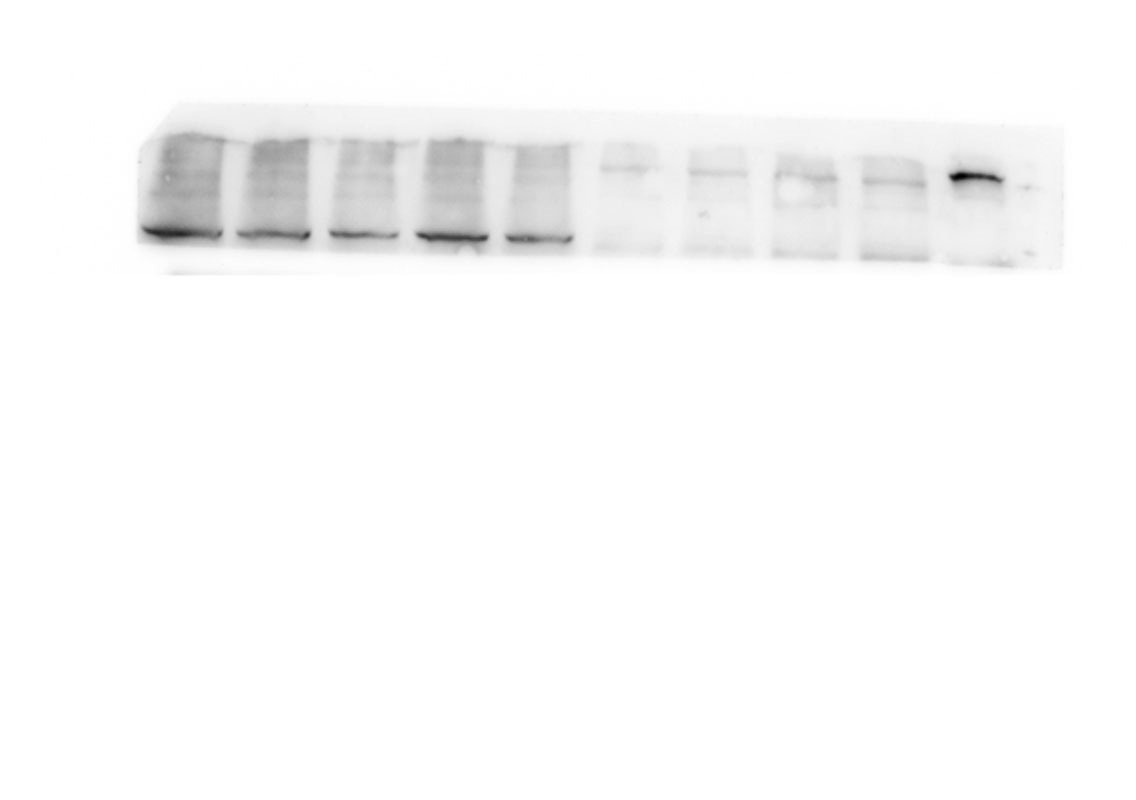

Supplement: Supplemental Information 1 [file peerj-11-16170-s001.zip › WB picture/Figure 6/CDKN1B.jpg]

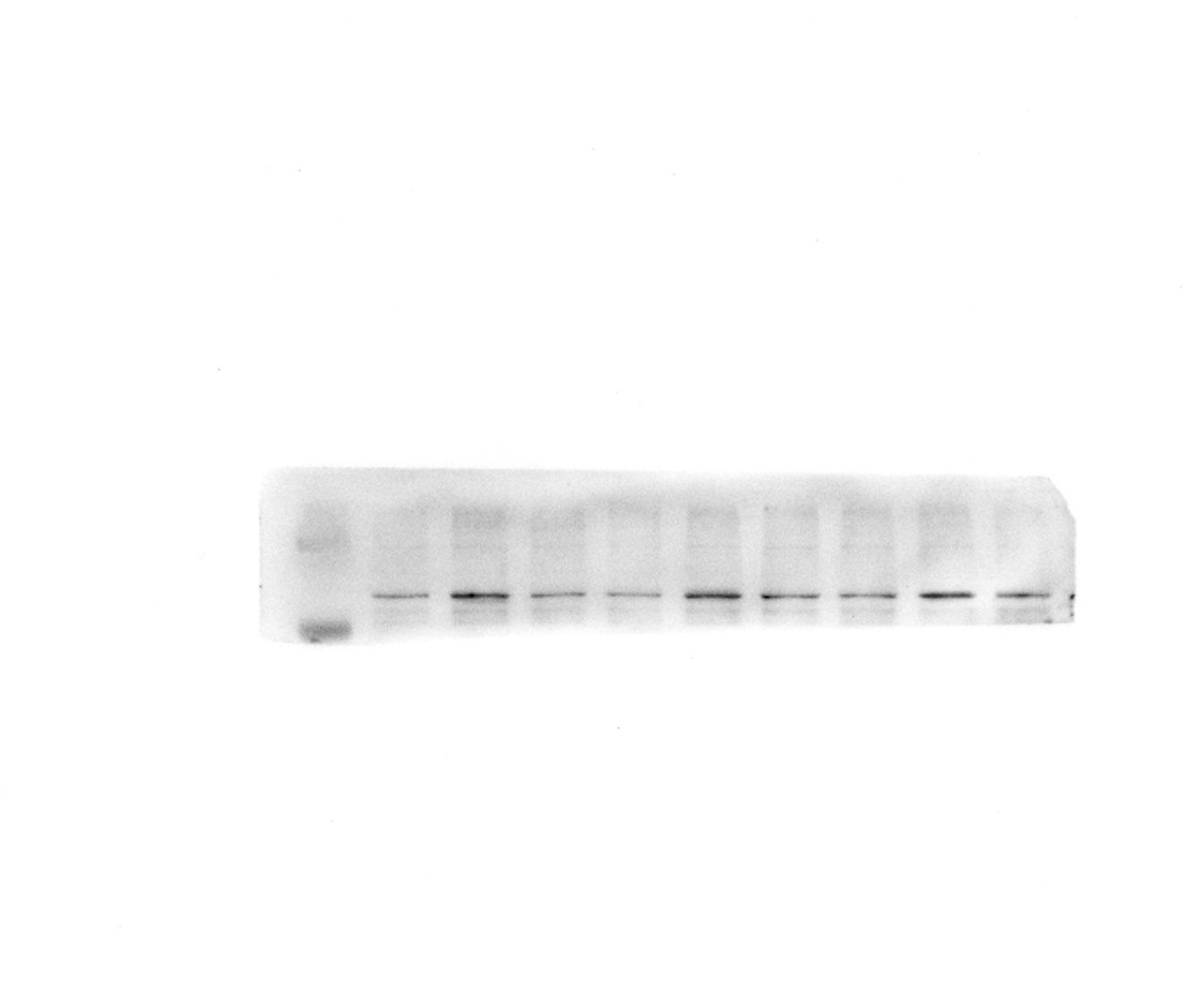

Supplement: Supplemental Information 1 [file peerj-11-16170-s001.zip › WB picture/Figure 2-4-5/Fig 2,4,5-Col IV.jpg]

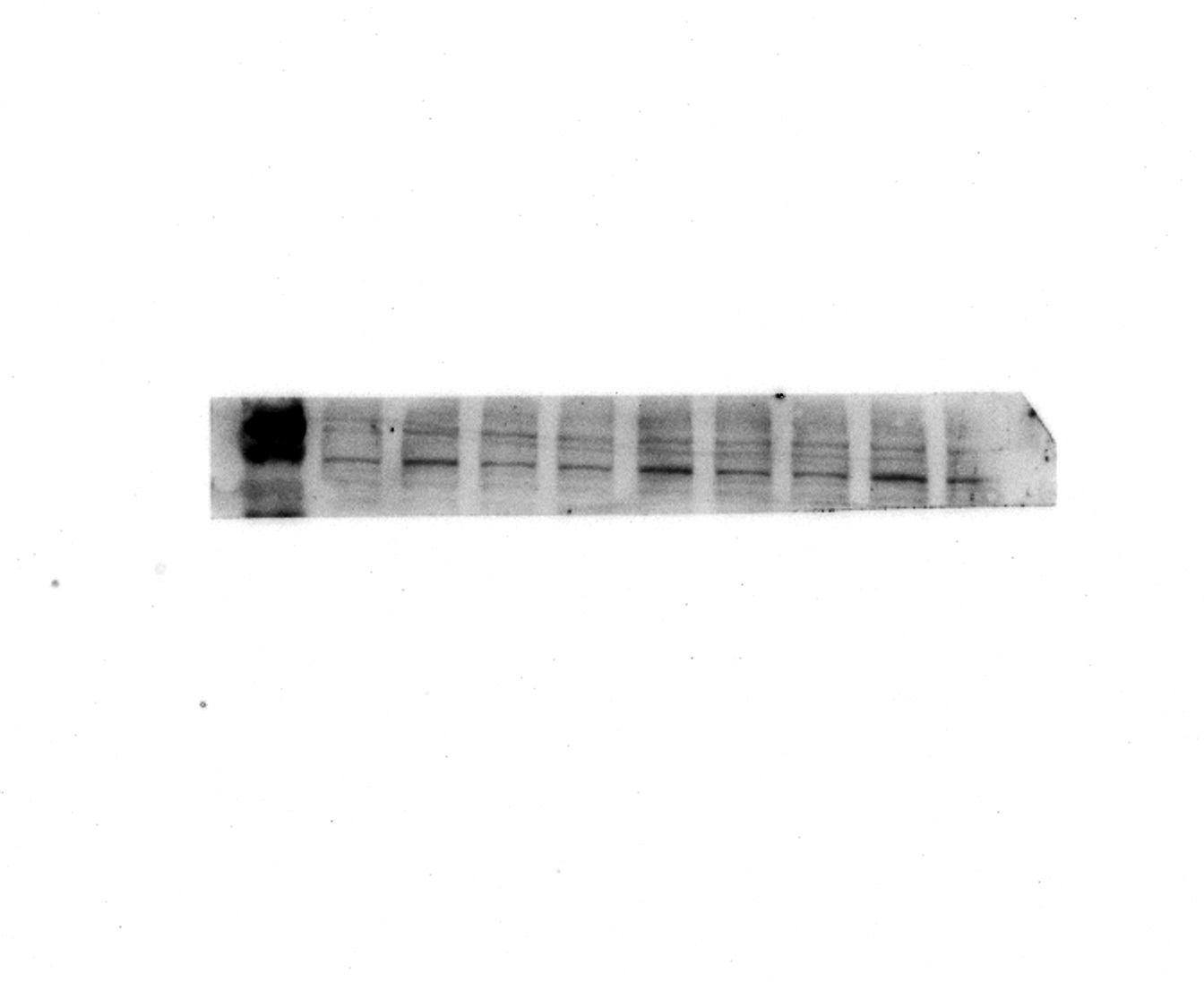

Supplement: Supplemental Information 1 [file peerj-11-16170-s001.zip › WB picture/Figure 2-4-5/Fig 2,4,5-FN.jpg]

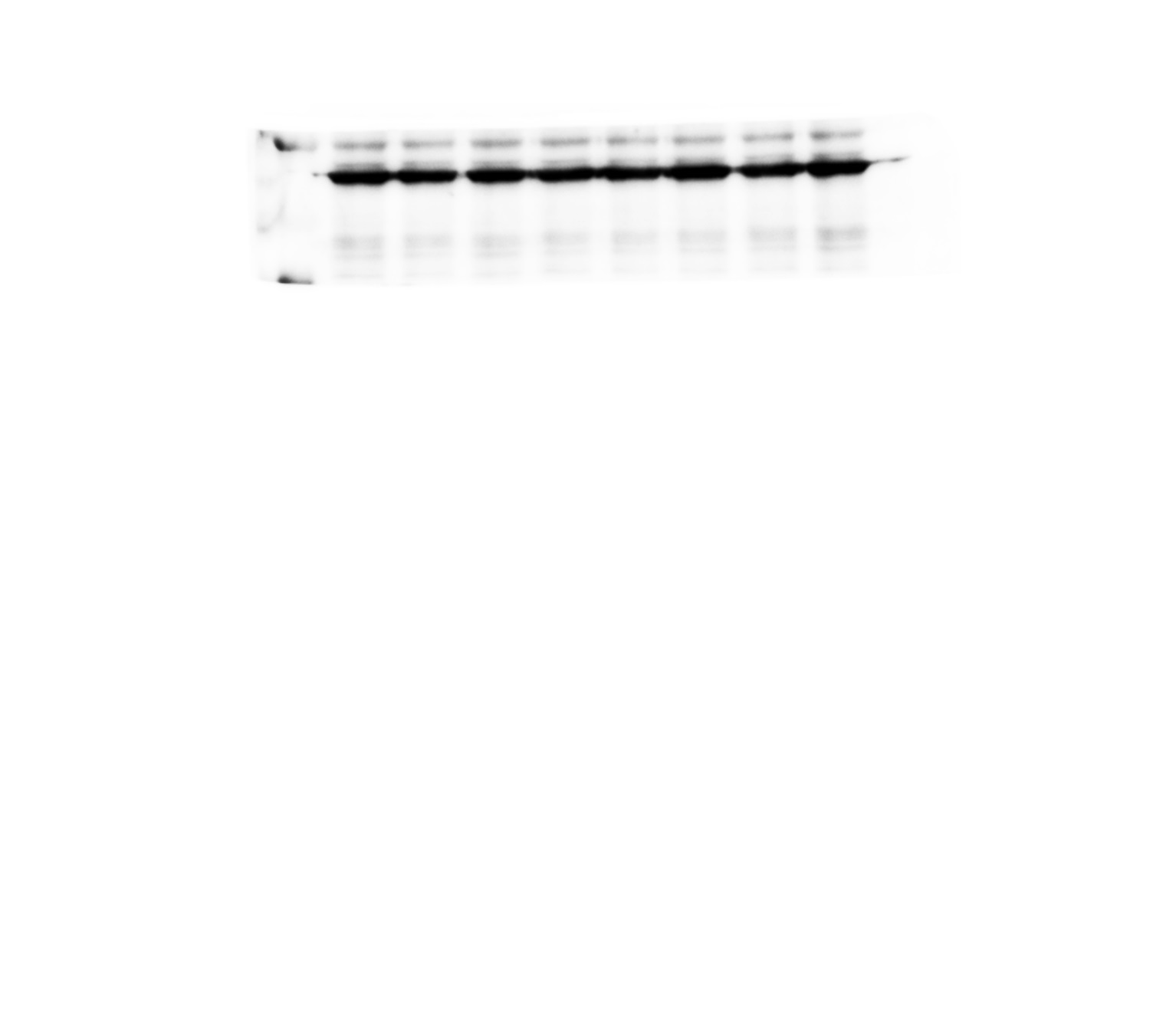

Supplement: Supplemental Information 1 [file peerj-11-16170-s001.zip › WB picture/Figure 6/BETA-ACTIN.jpg]

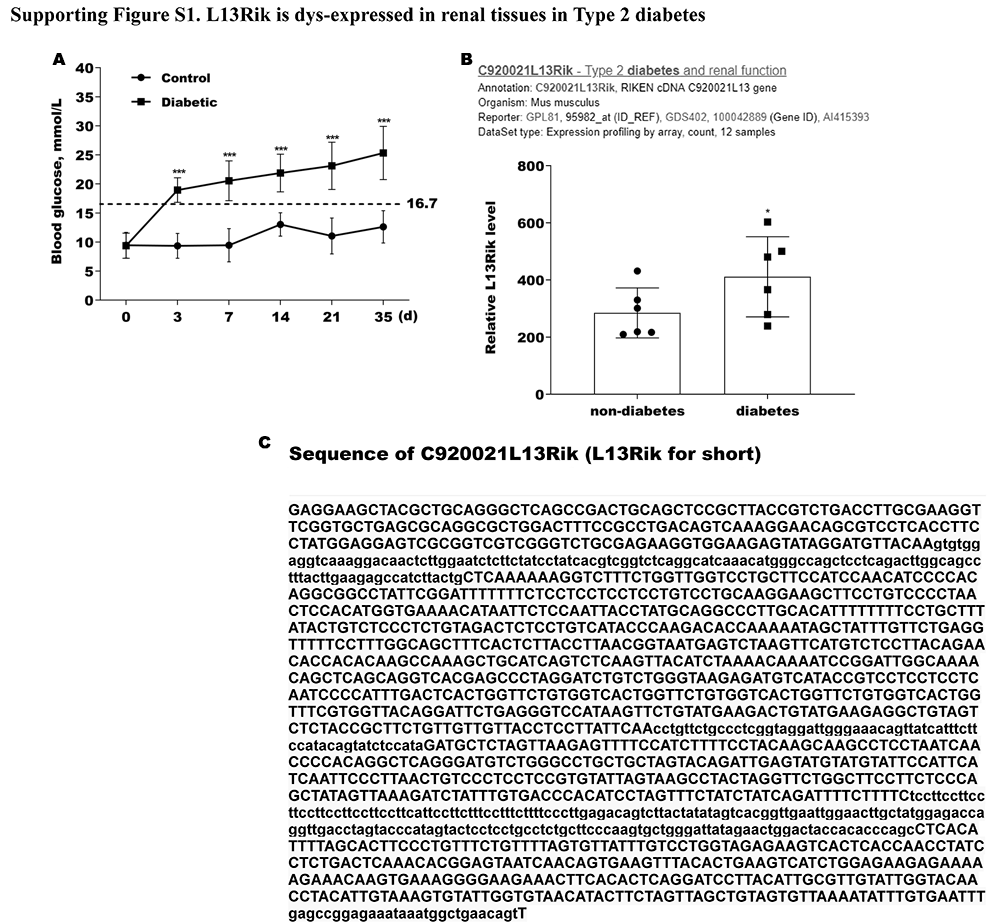

Supplement: Supplemental Information 5 [file peerj-11-16170-s005.png]

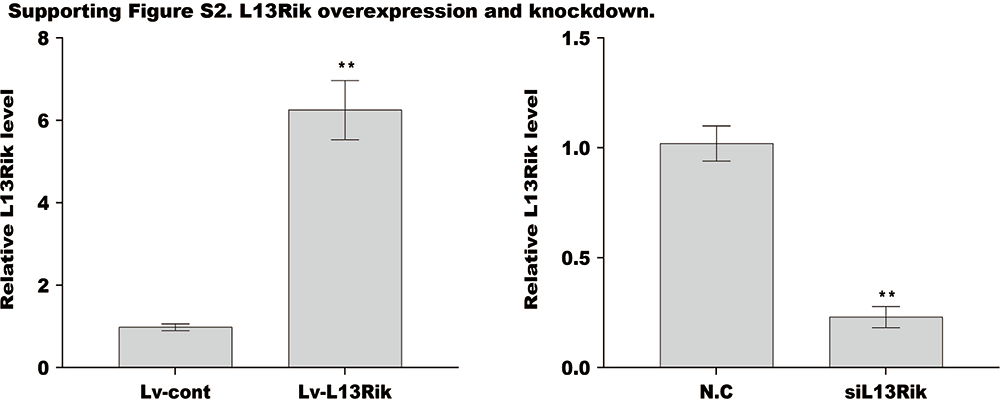

Supplement: Supplemental Information 6 [file peerj-11-16170-s006.png]

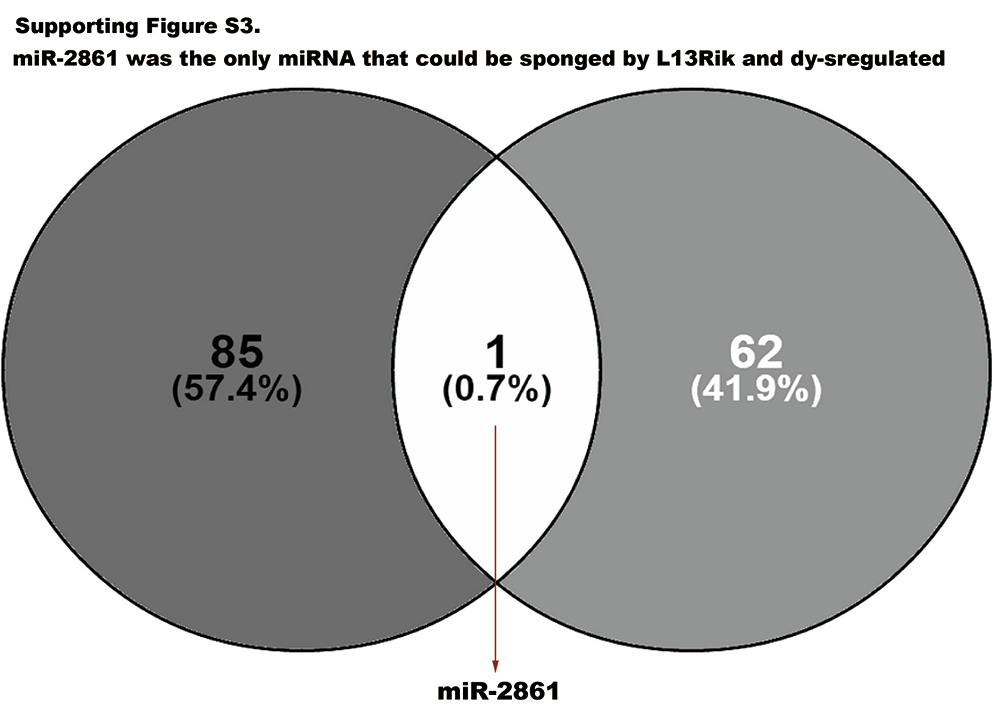

Supplement: Supplemental Information 7 [file peerj-11-16170-s007.png]
