# Supplementary material for: Long non‑coding RNA L13Rik promotes high glucose-induced mesangial cell hypertrophy and matrix protein expression by regulating miR-2861/CDKN1B axis
Source: PeerJ. 2023 Oct 16;11:e16170. doi: 10.7717/peerj.16170 (PMC10586299; doi:10.7717/peerj.16170)
Supplement: Supplemental Information 2 [file peerj-11-16170-s002.docx]

**Supporting Table S1.** **Sequences for qRT-PCR, RNAi, and miRNA**

| **Gene name** | **Primers sequences for qRT-PCR (5’-3')** |
| --- | --- |
| Mus musculus  GAPDH | Sense: GGAGAAACCTGCCAAGTATGA  Antisense: TCCTCAGTGTAGCCCAAGA |
| Homo sapiens  GAPDH | Sense: GGGAAGGTGAAGGTCGGAGT  Antisense: AGTTGAGGTCAATGAAGGGGTC |
| Mus musculus  L13Rik | Sense: CCTACAAGCAAGCCTCCTAATC  Antisense: GGAGGGACAGTTAAGGGAATTG |
| Homo sapiens  L13Rik | Sense: CTACAAGCAAGCCTCCTACC  Antisense: GTTAACTCTATGGACCAAATGC |
| Mus musculus  FN | Sense: CCTATTTTTGAAGATTTTGTGGAC  Antisense: TAATGAGAGTGATAACGCTGATGT |
| Mus musculus  Col IV | Sense: CAAGGTTCACCAGGGCTTAT  Antisense: GGGTCTCCTTTGTCACCTTT |
| Mus musculus  N-cad | Sense: GGATGAAACGGCGGGAT  Antisense: TCTTCTTCTCCTCCACCTTCTT |
| Mus musculus and  Homo sapiens U6 | Sense: CTCGCTTCGGCAGCACA  Antisense: AACGCTTCACGAATTTGCGT |
| Mus musculus  miR-2861 | RT:GTCGTATCCAGTGCAGGGTCCGAGGTATTCGCACTGGATACGACCCGCCC  Sense: CGCGGGGGCCTGGCGGC  Antisense: AGTGCAGGGTCCGAGGTATT |
| Homo sapiens  miR-2861 | RT:GTCGTATCCAGTGCAGGGTCCGAGGTATTCGCACTGGATACGACCCGCCC  Sense: CGCGGGGGCCTGGCGGT  Antisense: AGTGCAGGGTCCGAGGTATT |
| **Gene name** | **Sequences for miRNA** |
| Mus musculus  miR-2861 | GGGGCCUGGCGGCGGGCGG |
| **Gene name** | **Sequences for siRNA** |
| Mus musculus  L13Rik | AUACACUUUACAAUGUAGGUU |
