# Supplementary material for: Long non‑coding RNA L13Rik promotes high glucose-induced mesangial cell hypertrophy and matrix protein expression by regulating miR-2861/CDKN1B axis
Source: PeerJ. 2023 Oct 16;11:e16170. doi: 10.7717/peerj.16170 (PMC10586299; doi:10.7717/peerj.16170)
Supplement: Supplemental Information 3 [file peerj-11-16170-s003.docx]

**Supporting Table S2.** Potential miRNAs possibly sponged by L13Rik using miRDB (http://mirdb.org/cgi-bin/search.cgi.).

| **Target Detail** | **Target Rank** | **Target Score** | **miRNA Name** | **Gene Symbol** |
| --- | --- | --- | --- | --- |
|  | 1 | 100 | [hsa-miR-6876-5p](http://mirdb.org/cgi-bin/mature_mir.cgi?name=hsa-miR-6876-5p) | submission |
|  | 2 | 100 | [hsa-miR-4476](http://mirdb.org/cgi-bin/mature_mir.cgi?name=hsa-miR-4476) | submission |
|  | 3 | 94 | [hsa-miR-6892-5p](http://mirdb.org/cgi-bin/mature_mir.cgi?name=hsa-miR-6892-5p) | submission |
|  | 4 | 92 | [hsa-miR-4719](http://mirdb.org/cgi-bin/mature_mir.cgi?name=hsa-miR-4719) | submission |
|  | 5 | 92 | [hsa-miR-765](http://mirdb.org/cgi-bin/mature_mir.cgi?name=hsa-miR-765) | submission |
|  | 6 | 92 | [hsa-miR-11181-3p](http://mirdb.org/cgi-bin/mature_mir.cgi?name=hsa-miR-11181-3p) | submission |
|  | 7 | 92 | [hsa-miR-1252-5p](http://mirdb.org/cgi-bin/mature_mir.cgi?name=hsa-miR-1252-5p) | submission |
|  | 8 | 91 | [hsa-miR-4533](http://mirdb.org/cgi-bin/mature_mir.cgi?name=hsa-miR-4533) | submission |
|  | 9 | 89 | [hsa-miR-8081](http://mirdb.org/cgi-bin/mature_mir.cgi?name=hsa-miR-8081) | submission |
|  | 10 | 86 | [hsa-miR-6165](http://mirdb.org/cgi-bin/mature_mir.cgi?name=hsa-miR-6165) | submission |
|  | 11 | 86 | [hsa-miR-937-5p](http://mirdb.org/cgi-bin/mature_mir.cgi?name=hsa-miR-937-5p) | submission |
|  | 12 | 83 | [hsa-miR-589-3p](http://mirdb.org/cgi-bin/mature_mir.cgi?name=hsa-miR-589-3p) | submission |
|  | 13 | 82 | [hsa-miR-4428](http://mirdb.org/cgi-bin/mature_mir.cgi?name=hsa-miR-4428) | submission |
|  | 14 | 82 | [hsa-miR-3937](http://mirdb.org/cgi-bin/mature_mir.cgi?name=hsa-miR-3937) | submission |
|  | 15 | 82 | [hsa-miR-6124](http://mirdb.org/cgi-bin/mature_mir.cgi?name=hsa-miR-6124) | submission |
|  | 16 | 80 | [hsa-miR-4531](http://mirdb.org/cgi-bin/mature_mir.cgi?name=hsa-miR-4531) | submission |
|  | 17 | 77 | [hsa-miR-708-3p](http://mirdb.org/cgi-bin/mature_mir.cgi?name=hsa-miR-708-3p) | submission |
|  | 18 | 76 | [hsa-miR-3148](http://mirdb.org/cgi-bin/mature_mir.cgi?name=hsa-miR-3148) | submission |
|  | 19 | 75 | [hsa-miR-8055](http://mirdb.org/cgi-bin/mature_mir.cgi?name=hsa-miR-8055) | submission |
|  | 20 | 75 | [hsa-miR-196a-1-3p](http://mirdb.org/cgi-bin/mature_mir.cgi?name=hsa-miR-196a-1-3p) | submission |
|  | 21 | 74 | [hsa-miR-1286](http://mirdb.org/cgi-bin/mature_mir.cgi?name=hsa-miR-1286) | submission |
|  | 22 | 74 | [hsa-miR-6847-5p](http://mirdb.org/cgi-bin/mature_mir.cgi?name=hsa-miR-6847-5p) | submission |
|  | 23 | 73 | [hsa-miR-8065](http://mirdb.org/cgi-bin/mature_mir.cgi?name=hsa-miR-8065) | submission |
|  | 24 | 70 | [hsa-miR-22-3p](http://mirdb.org/cgi-bin/mature_mir.cgi?name=hsa-miR-22-3p) | submission |
|  | 25 | 69 | [hsa-miR-4316](http://mirdb.org/cgi-bin/mature_mir.cgi?name=hsa-miR-4316) | submission |
|  | 26 | 69 | [hsa-miR-6133](http://mirdb.org/cgi-bin/mature_mir.cgi?name=hsa-miR-6133) | submission |
|  | 27 | 69 | [hsa-miR-6130](http://mirdb.org/cgi-bin/mature_mir.cgi?name=hsa-miR-6130) | submission |
|  | 28 | 69 | [hsa-miR-6129](http://mirdb.org/cgi-bin/mature_mir.cgi?name=hsa-miR-6129) | submission |
|  | 29 | 69 | [hsa-miR-6127](http://mirdb.org/cgi-bin/mature_mir.cgi?name=hsa-miR-6127) | submission |
|  | 30 | 69 | [hsa-miR-4510](http://mirdb.org/cgi-bin/mature_mir.cgi?name=hsa-miR-4510) | submission |
|  | 31 | 66 | [hsa-miR-323b-3p](http://mirdb.org/cgi-bin/mature_mir.cgi?name=hsa-miR-323b-3p) | submission |
|  | 32 | 65 | [hsa-miR-3157-5p](http://mirdb.org/cgi-bin/mature_mir.cgi?name=hsa-miR-3157-5p) | submission |
|  | 33 | 64 | [hsa-miR-4779](http://mirdb.org/cgi-bin/mature_mir.cgi?name=hsa-miR-4779) | submission |
|  | 34 | 64 | [hsa-miR-302d-5p](http://mirdb.org/cgi-bin/mature_mir.cgi?name=hsa-miR-302d-5p) | submission |
|  | 35 | 64 | [hsa-miR-302b-5p](http://mirdb.org/cgi-bin/mature_mir.cgi?name=hsa-miR-302b-5p) | submission |
|  | 36 | 63 | [hsa-miR-4455](http://mirdb.org/cgi-bin/mature_mir.cgi?name=hsa-miR-4455) | submission |
|  | 37 | 63 | [hsa-miR-1297](http://mirdb.org/cgi-bin/mature_mir.cgi?name=hsa-miR-1297) | submission |
|  | 38 | 61 | [hsa-miR-574-5p](http://mirdb.org/cgi-bin/mature_mir.cgi?name=hsa-miR-574-5p) | submission |
|  | 39 | 60 | [hsa-miR-4266](http://mirdb.org/cgi-bin/mature_mir.cgi?name=hsa-miR-4266) | submission |
|  | 40 | 59 | [hsa-miR-5197-3p](http://mirdb.org/cgi-bin/mature_mir.cgi?name=hsa-miR-5197-3p) | submission |
|  | 41 | 59 | [hsa-miR-6500-3p](http://mirdb.org/cgi-bin/mature_mir.cgi?name=hsa-miR-6500-3p) | submission |
|  | 42 | 58 | [hsa-miR-6857-5p](http://mirdb.org/cgi-bin/mature_mir.cgi?name=hsa-miR-6857-5p) | submission |
|  | 43 | 58 | [hsa-miR-2861](http://mirdb.org/cgi-bin/mature_mir.cgi?name=hsa-miR-2861) | submission |
|  | 44 | 58 | [hsa-miR-4319](http://mirdb.org/cgi-bin/mature_mir.cgi?name=hsa-miR-4319) | submission |
|  | 45 | 57 | [hsa-miR-7161-3p](http://mirdb.org/cgi-bin/mature_mir.cgi?name=hsa-miR-7161-3p) | submission |
|  | 46 | 57 | [hsa-miR-5702](http://mirdb.org/cgi-bin/mature_mir.cgi?name=hsa-miR-5702) | submission |
|  | 47 | 56 | [hsa-miR-4784](http://mirdb.org/cgi-bin/mature_mir.cgi?name=hsa-miR-4784) | submission |
|  | 48 | 56 | [hsa-miR-3150b-3p](http://mirdb.org/cgi-bin/mature_mir.cgi?name=hsa-miR-3150b-3p) | submission |
|  | 49 | 55 | [hsa-miR-4516](http://mirdb.org/cgi-bin/mature_mir.cgi?name=hsa-miR-4516) | submission |
|  | 50 | 53 | [hsa-miR-135a-2-3p](http://mirdb.org/cgi-bin/mature_mir.cgi?name=hsa-miR-135a-2-3p) | submission |
|  | 51 | 53 | [hsa-miR-1225-5p](http://mirdb.org/cgi-bin/mature_mir.cgi?name=hsa-miR-1225-5p) | submission |
|  | 52 | 53 | [hsa-miR-6891-5p](http://mirdb.org/cgi-bin/mature_mir.cgi?name=hsa-miR-6891-5p) | submission |
|  | 53 | 53 | [hsa-miR-4426](http://mirdb.org/cgi-bin/mature_mir.cgi?name=hsa-miR-4426) | submission |
|  | 54 | 53 | [hsa-miR-5584-5p](http://mirdb.org/cgi-bin/mature_mir.cgi?name=hsa-miR-5584-5p) | submission |
|  | 55 | 52 | [hsa-miR-12131](http://mirdb.org/cgi-bin/mature_mir.cgi?name=hsa-miR-12131) | submission |
|  | 56 | 52 | [hsa-miR-620](http://mirdb.org/cgi-bin/mature_mir.cgi?name=hsa-miR-620) | submission |
|  | 57 | 52 | [hsa-miR-1270](http://mirdb.org/cgi-bin/mature_mir.cgi?name=hsa-miR-1270) | submission |
|  | 58 | 52 | [hsa-miR-302e](http://mirdb.org/cgi-bin/mature_mir.cgi?name=hsa-miR-302e) | submission |
|  | 59 | 52 | [hsa-miR-4536-5p](http://mirdb.org/cgi-bin/mature_mir.cgi?name=hsa-miR-4536-5p) | submission |
|  | 60 | 52 | [hsa-miR-6515-3p](http://mirdb.org/cgi-bin/mature_mir.cgi?name=hsa-miR-6515-3p) | submission |
|  | 61 | 51 | [hsa-miR-4781-3p](http://mirdb.org/cgi-bin/mature_mir.cgi?name=hsa-miR-4781-3p) | submission |
|  | 62 | 51 | [hsa-miR-6505-3p](http://mirdb.org/cgi-bin/mature_mir.cgi?name=hsa-miR-6505-3p) | submission |
|  | 63 | 50 | [hsa-miR-7844-5p](http://mirdb.org/cgi-bin/mature_mir.cgi?name=hsa-miR-7844-5p) | submission |
