# Supplementary material for: Long non‑coding RNA L13Rik promotes high glucose-induced mesangial cell hypertrophy and matrix protein expression by regulating miR-2861/CDKN1B axis
Source: PeerJ. 2023 Oct 16;11:e16170. doi: 10.7717/peerj.16170 (PMC10586299; doi:10.7717/peerj.16170)
Supplement: Supplemental Information 4 [file peerj-11-16170-s004.docx]

**Supporting Table S3.** Altered or dysregulated miRNAs reported in human DN studies(1).

| miRNA | Upregulated | Downregulated |
| --- | --- | --- |
| let-7a-5p |  | √ |
| miR-10 |  | √ |
| miR-100-5p | √ |  |
| miR-1224-3p | √ |  |
| miR-122-5p | √ |  |
| miR-1247-5p |  |  |
| miR-126 |  | √ |
| miR-126-3p | √ |  |
| miR-1303 | √ |  |
| miR-130a | √ |  |
| miR-130b | √ | √ |
| miR-130b-3p |  |  |
| miR-135a-5p | √ |  |
| miR-141-3p | √ |  |
| miR-145 | √ |  |
| miR-146a | √ |  |
| miR-155 | √ |  |
| miR-17-5p | √ |  |
| miR-181a | √ |  |
| miR-186-5p | √ |  |
| miR-1912 | √ |  |
| miR-1915-3p | √ | √ |
| miR-192 |  | √ |
| miR-199a-5p | √ |  |
| miR-200b-3p |  | √ |
| miR-200a-3p |  | √ |
| miR-200c-3p |  | √ |
| miR-21-5p | √ |  |
| miR-214-3p | √ |  |
| miR-215 | √ |  |
| miR-21 | √ |  |
| miR-216a |  | √ |
| miR-221-3p |  | √ |
| miR-23 |  |  |
| miR-23b |  | √ |
| miR-25-3p |  | √ |
| miR-26a-5p |  | √ |
| miR-27a | √ |  |
| miR-2861 |  | √ |
| miR-29a-3p |  | √ |
| miR-29b-1-5p | √ |  |
| miR-29c | √ |  |
| miR-29c-3p |  | √ |
| miR-30 |  |  |
| miR-302a-3p | 2√ |  |
| miR-30a |  | √ |
| miR-30a-5p | √ |  |
| miR-30c-5p | √ |  |
| miR-30d-5p |  | √ |
| miR-30e-5p |  | √ |
| miR-320a | √ |  |
| miR-320c | √ |  |
| miR-323b-5p |  | √ |
| miR-326 | √ |  |
| miR-335-5p | √ |  |
| miR-377 | √ |  |
| miR-424 |  | √ |
| miR-424-5p | √ |  |
| miR-4270 | √ |  |
| miR-429 | √ |  |
| miR-4532 |  | √ |
| miR-4536-3p | √ |  |
| miR-486-3p | √ |  |
| miR-495 | √ |  |
| miR-495-3p |  | √ |
| miR-501-5p | √ |  |
| miR-548c-3p | √ |  |
| miR-548o-3p | √ |  |
| miR-552 | √ |  |
| miR-571 | √ |  |
| miR-574-3p |  | √ |
| miR-6068 | √ |  |
| miR-616-5p | √ |  |
| miR-619 | √ |  |
| miR-640 | √ |  |
| miR-645 | √ |  |
| miR-661 | √ |  |
| miR-665 | √ |  |
| miR-6747 | √ |  |
| miR-767-3p | √ |  |
| miR-770-5p | √ |  |
| miR-7a-5p | √ |  |
| miR-7b-3p | √ |  |
| miR-886 |  | √ |
| miR-892b | √ |  |
| miR-93-5p | √ |  |

1. Chen, L., Wu, B., Wang, S., Xiong, Y., Zhou, B., Cheng, X., Zhou, T., Luo, R., Lam, T.W., Yan, B., et al. 2020. Identification of Cooperative Gene Regulation Among Transcription Factors, LncRNAs, and MicroRNAs in Diabetic Nephropathy Progression. *Front Genet* 11:1008.
