# Supplementary material for: Long non‑coding RNA L13Rik promotes high glucose-induced mesangial cell hypertrophy and matrix protein expression by regulating miR-2861/CDKN1B axis
Source: PeerJ. 2023 Oct 16;11:e16170. doi: 10.7717/peerj.16170 (PMC10586299; doi:10.7717/peerj.16170)
Supplement: Supplemental Information 8 — There are three repetitions in the file, Repeat 1 are used for main figures. [file peerj-11-16170-s008.pdf]

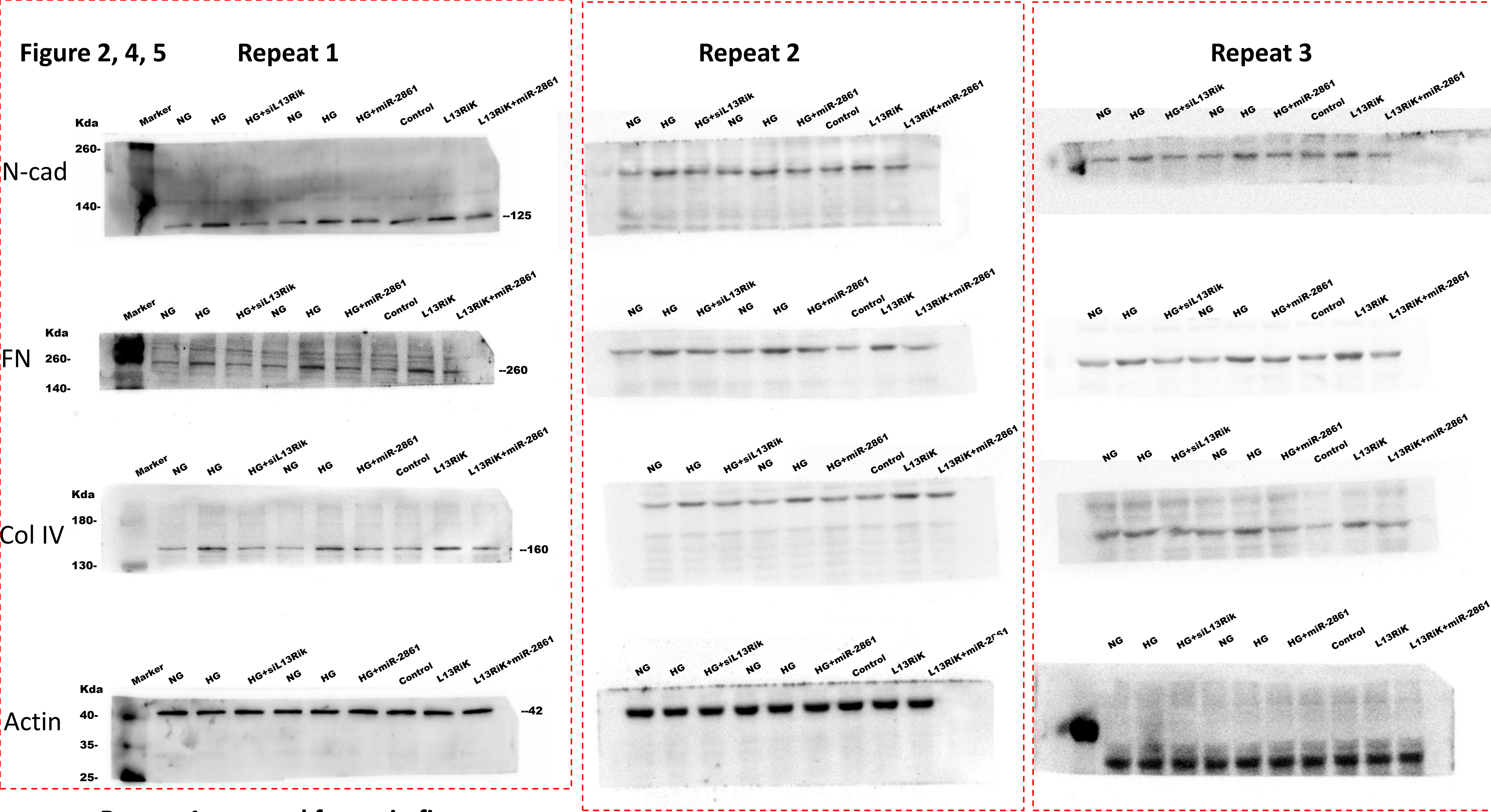

Repeat 1 are used for main figures

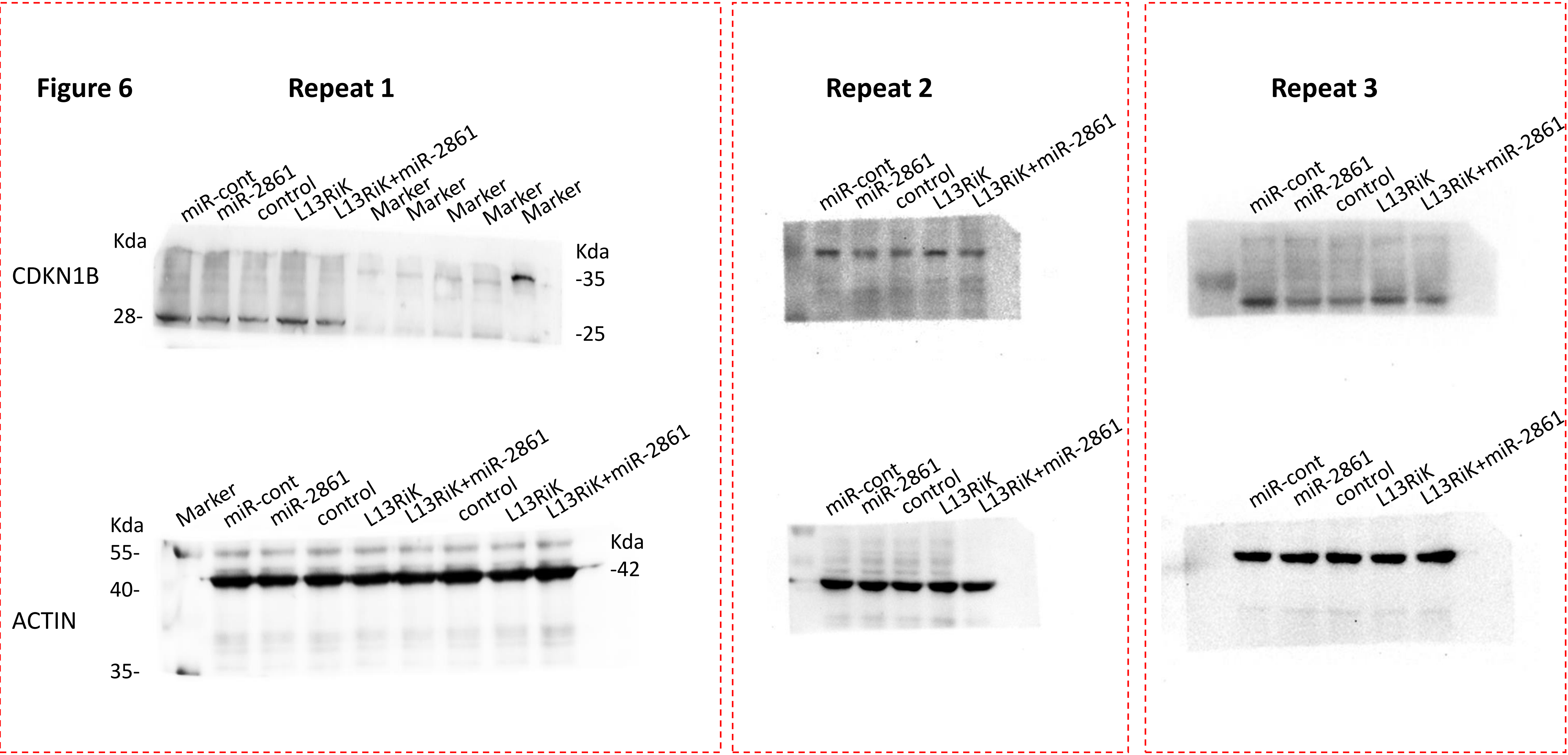

Repeat 1 are used for main figures
